# Supplementary material for: Chromothripsis during telomere crisis is independent of NHEJ, and consistent with a replicative origin
Source: Genome Res. 2019 May;29(5):737–49. doi: 10.1101/gr.240705.118 (PMC6499312; doi:10.1101/gr.240705.118)
Supplement: Supplemental Material [file supp_gr.240705.118_Supplemental_file_1.zip › contigs/annotated_contigs/DB73/contig.2.DB73_length_622_mean_cov_6.27009646302.docx]

**DB73_length_622_mean_cov_6.27009646302**

```
AGGATTAAGAGAGTAGTTGAAAATTTAGAAGGGAAAGATGATAATTGATACAGCAAAGTTTTTGAGGAGGCAAGTGATAGAGTGTAGTG
 >chrX:37581602-37581961 + E=2e-204
CAGGTGGGGAGATCCCGGCTCCAGATCGTAAGAAAGACTCTTTGTTCACTGAGTTGAAAGAAAACAGATAAAAATTACTGTCTCTCAGA

AATCTGTTGAAAATTCTAATTTTCAGAAGCTTGCTTTGCTAACTTCCTCCAGTTTCCCTCTGCCACTGCACTCCATATCCCCTTTGGCT

TCGCCAGTTACCTCTTTGAAGTCATTCACTAATTTTTTTGAAAAGTCTATTCCATAAAAGCATTTCATGAGTTCAGTCATTTTACTG|A

AGAT|CGTCTCTAGGAACCTGTCCTTACCTCCCAGAAAAACTGCCTACAAAATATATTCCTCTGTAACTGTAAGTAATTCAGGGCAAAG
>chrX:37544195-37544463 - E=1e-149
ATCAGGGCCTAATAAGGAAGTAGTTTTTTTTATTCCAAGACAGACCTCTGCTTATTGGTGTGCCAATACCAAGACTAACACAACTCCTT

TTAGCCCATTGAACTTTCCACCATCTTCTTGATATCAGGATGTACTCAGTCCACCCCCGTCCCTCAGACCTCGATCAAGTCACTAAATC

T
```
